# The effect of door-to-balloon delay in primary percutaneous coronary intervention on clinical outcomes of STEMI: a systematic review and meta-analysis protocol

**DOI:** 10.1186/s13643-016-0304-7

**Published:** 2016-08-02

**Authors:** Chee Yoong Foo, Daniel D. Reidpath, Nathorn Chaiyakunapruk

**Affiliations:** 1 National Clinical Research Centre, Kuala Lumpur, Malaysia; 2 School of Pharmacy, Monash University Malaysia, Jalan Lagoon Selatan, Bandar Sunway, 47500 Selangor Darul Ehsan Malaysia; 3 Jeffrey Cheah School of Medicine and Health Sciences, Monash University Malaysia, Jalan Lagoon Selatan, Bandar Sunway, 47500 Selangor Darul Ehsan Malaysia; 4 Center of Pharmaceutical Outcomes Research (CPOR), Department of Pharmacy Practice, Faculty of Pharmaceutical Sciences, Naresuan University, Phitsanulok, Thailand; 5 School of Pharmacy, University of Wisconsin, Madison, WI USA; 6 School of Population Health, University of Queensland, Brisbane, Australia

**Keywords:** Door-to-balloon time, STEMI, Percutaneous coronary intervention, Protocol, Systematic review

## Abstract

**Background:**

Acute myocardial infarction (AMI) is a medical emergency in which sudden occlusion of coronary artery(ies) results in ischemia and necrosis of the cardiac tissues. Reperfusion therapies that aim at reopening the occluded artery remain the mainstay of treatment for AMI. Primary percutaneous coronary intervention (PCI), which enables the restoration of blood flow by reopening the occluded artery(ies) via a catheter with an inflatable balloon, is currently the preferred treatment for AMI with ST segment elevation (STEMI). The door-to-balloon (D2B) delay refers to the time interval counting from the arrival of a patient with STEMI at a hospital to the time of the balloon inflation (or stent deployment) that reopens the occluded artery(ies). Reducing this delay in primary PCI is thought to be an important strategy toward achieving better patient outcomes. Unfortunately, significant reduction of D2B delay in the USA over the last decade has not been shown to be associated with improved STEMI mortality. It has been suggested that the lack of impact could be due to the expanding use of primary PCI in STEMI as well as the survival cohort effect, leading to a shift toward a higher risk population receiving the procedure. Others have suggested that reduction in D2B delay may not be as impactful as expected, given that it only represents a small fraction of the total ischemic time. Although most existing evidence have pointed to the presence of a beneficial effect of shorter D2B delay, some inconsistencies however exist. This study aims to synthesize available evidence in order to answer the following questions: (1) what is the overall effect of D2B delay on clinical outcomes in patients with STEMI treated with primary PCI? (2) What factors explain the differences of the effect estimates among the studies? (3) What are the important strength and limitation of the existing body of evidence?

**Method:**

We will search PubMed/MEDLINE, EMBASE, ClinicalTrials.gov, WHO International Clinical Trials Registry, CINAHL Database, and the Cochrane Library using a predefined search strategy. Other sources of literature will include proceedings from the European Society of Cardiology, the American College of Cardiology, the American Heart Association, the EUROPCR, and the ProQuest Dissertations and Theses Database. We will include data from observational studies (case-control and cohort study design) and randomized control trials (that have investigated the relationship of D2B time and clinical outcome(s) in an adult (older than 18) STEMI population). Mortality (cardiac related and all-cause) and incidence heart failure (HF) have been prioritized as the primary outcomes. All eligible studies will be assessed for risk of bias using the Risk Of Bias in Non-randomized Studies - of Interventions tool. The Grading of Recommendations, Assessment, and Evaluation (GRADE) framework will be used to report the quality of evidence and strength of recommendations. We will proceed to analyze the data quantitatively if the pre-specified conditions are satisfied.

**Discussion:**

Recent discussion on the negative findings of improved D2B delay over time being unrelated to better STEMI outcomes at the population level has reminded us of an important knowledge gap we have on this domain. This systematic review will serve to address some of these key questions not previously examined. Answers to these questions could clarify the controversies and offer empirical support for or against the suggested hypotheses.

**Systematic review registration:**

PROSPERO CRD42015026069

**Electronic supplementary material:**

The online version of this article (doi:10.1186/s13643-016-0304-7) contains supplementary material, which is available to authorized users.

## Introduction

### Background

Acute myocardial infarction (AMI) is a medical emergency in which sudden occlusion of the coronary artery(ies) results in ischemia and necrosis of the cardiac tissues [1]. It is one of the leading causes of mortality and morbidity worldwide with an estimated global incidence of 8.5 million in 2013 [2].

Reperfusion therapies that aim at reopening the occluded artery remain the mainstay of treatment for AMI [3]. In order to decide if urgent reperfusion is needed, clinicians designate cases of AMI by the presence or absence of an important electrocardiogram (ECG) finding—the ST segment elevation. This ECG feature, if present, signifies the presence of cardiac cell death. Primary percutaneous coronary intervention (PCI)—a mechanical intervention that enables the restoration of blood flow by reopening the occluded artery via a catheter with an inflatable balloon—is currently the preferred reperfusion option for AMI with ST segment elevation (hereafter STEMI) [3].

The door-to-balloon (D2B) delay refers to the time interval counting from the arrival of a patient with STEMI at a hospital to the time of the balloon inflation (or stent deployment) that reopens the occluded artery(ies) [4]. It constitutes part of the total time interval from the onset of the occlusion to the reopening of the blocked artery(ies) (this time interval is commonly known as the total ischemic time (TIT)). Reducing this delay shortens the TIT and hence is thought to be an important strategy toward achieving better patient outcomes. The idea of its importance was first suggested by the open artery theory [5] developed through animal experiments [6, 7] and supported by human angiographic studies [8]; empirical evidence from analyses of controlled trial data [9] and registry data [10, 11] have provided additional strength to its independent effect on STEMI outcomes. Based on these evidence, contemporary practice guidelines [3] recommend a 90-min target for D2B time as a strategy to achieve high-quality reperfusion. As a result of the recommendation, significant resources have been invested in reducing such delay across healthcare systems globally for the past decades. The D2B Alliance [12] conceived in 2006 by the American College of Cardiology is perhaps the most well-received example.

Although the D2B delay has improved significantly over the past decades in many healthcare systems [13, 14], recent assessment of its impact on the mortality rate for those with STEMI undergoing primary PCI has disappointed many [15, 16]. Improvement of the national D2B times in the USA has not changed the in-hospital mortality rate for the population of patients undergoing primary PCI [16]. It has been suggested that the lack of impact could be due to the expanding use of primary PCI in STEMI as well as the alteration of the survival cohort effect over time, leading to a shift toward a higher risk STEMI population receiving the procedure [17]. Benefit gained from the improved D2B delay could have been offset by such a move. Others have suggested that reduction in D2B delay may not be as impactful as expected, given that it only represents a small fraction of the total ischemic time [16].

While most evidence so far have pointed to the presence of an effect of D2B delay on STEMI outcomes, some inconsistencies exist. Not all studies found the presence of an effect; some suggested a significant effect only among those presented to the hospitals early after the symptom onset [9, 18]; those with high mortality risk appeared to be affected differently [19]. Given that a large proportion of the STEMI population presents late to the hospital after symptom onset [20, 21], it is possible that reducing the D2B delay in real life may not be as effective as previously thought in reducing STEMI mortality.

Earlier reviews [22, 23] on this topic have synthesized useful insights, yet weaknesses in their conducts, including suboptimal search strategies, lack of comprehensive risk of bias assessment, and incomplete quantitative analyses have limited our ability to draw conclusions with confidence. For instance, Chen [22] searched only PubMed and ISI databases and imposed a language (English) restriction in their search strategy; risk of bias assessment was not performed in both of these reviews. Moreover, analyses that are potentially insightful to some important questions have not been attempted. For example, the hypothesis that dissimilarity of risk level among different study population played a role in the differences of the estimated effect size has yet to be tested above the level of individual studies. This review will address the methodological weaknesses of the previous ones as well as attempt to answer some important questions (described below) not previously attempted.

### Study objectives

This study protocol will provide a road map and allow transparency in synthesizing the available evidence in order to answer the following questions:

1. What is the overall effect of D2B delay on clinical outcomes in patients with STEMI treated with primary PCI?
2. What factors explain the differences of the effect estimates among the studies?
3. What are the important strength and limitation of the existing body of evidence?

We hypothesize that

1. Shorter D2B delay in primary PCI is related to better outcomes overall;
2. The magnitude of effect estimates of D2B delay on STEMI outcomes is affected by
  - Patient population risk profile—studies conducted in higher risk population derived larger benefit;
  - Study sample size—smaller studies may yield larger effect size;
  - Degree of pre-hospital delay (pre-hospital delay refers to the time interval from symptom onset to hospital arrival)—effect size is larger among those with shorter pre-hospital delay
  - Study-level risk of bias—effect size is larger in studies that lack adequate risk adjustment.

When appropriate, we aim to answer these questions quantitatively. We will describe the criteria for decision to proceed with quantitative analysis in the section of data synthesis below.

## Methods

### Search strategy

We constructed the search strategy using the PICO framework. The search terms include controlled terms from MeSH in PubMed and The Cochrane Library and EMtree in EMBASE. The search strategy is provided in Additional file 1.

No language restriction will be applied. We will set up a search alert after the initial attempt to ensure that latest relevant literature will not be excluded. The first author of this protocol (FCY) will perform the search. This protocol follows the Preferred Reporting Items for Systematic Reviews and Meta-Analyses Protocol 2015 (PRISMA-P) (included as Additional file 2).

### Information sources

We will search PubMed/MEDLINE, EMBASE, ClinicalTrials.gov, WHO International Clinical Trials Registry, CINAHL Database, and The Cochrane Library from 1977 (year when the first angioplasty was performed [24]) to December 2015 for published data. Other sources will include proceedings from the European Society of Cardiology (www.escardio.org), the American College of Cardiology (www.acc.org), the American Heart Association (www.aha.org), the EUROPCR (www.europcr.com), and the TCT (www.tctconference.com) for the past 20 years. We also plan to search the ProQuest Dissertations and Theses Database for additional grey literature. Manual search will be conducted on the reference list of relevant articles. We will contact experts in the field to ensure that important publications are included.

### Eligibility criteria

We will include prospective observational studies (case-control and cohort study design) that have investigated the D2B time and clinical outcome relationship in adult (older than 18) STEMI population. Randomized control trials which have investigated the effect of interventions that aimed to shorten D2B time on clinical outcomes of STEMI will also be included if the investigators have analyzed the D2B time-outcome relationship. Eligible studies shall have compared at least one clinical outcome in between different patient groups of D2B time exposure. No demographic limitation will be applied. Both published and unpublished reports will be included where available.

### Outcomes and prioritization

Mortality reduction and heart failure prevention are still the most important goals of today’s STEMI care. Therefore, we will prioritize in this review mortality (cardiac related and all-cause) and incidence heart failure (HF) as the primary outcomes of study. All other clinical endpoints, including cardiac arrest (not resulting in death), tachy- and bradyarrhythmia, and cardiac wall aneurysm, will form the secondary outcomes.

We follow the definition of the European Society of Cardiology guideline for heart failure (HF) [25], which states that “HF is a syndrome in which patients have typical symptoms (e.g., breathlessness, ankle swelling, and fatigue) and signs (e.g., elevated jugular venous pressure, pulmonary crackles, and displaced apex beat) resulting from abnormal cardiac structure or function.”

### Data management

Duplicated records will first be removed from the set of retrieved articles using Endnotes reference management software. De-duplicated set of the retrieved citations and abstracts will then be imported to an article screening program built using Microsoft Access. Each screener will be provided with a complete set of the de-duplicated citations and abstracts. They will be able to perform the screening and coding within the screening program. Full text of all the selected articles will then be retrieved and examined in detail.

### Selection process

We will first develop, test, and refine the article screening form based on the pre-specified eligibility criteria stated above and perform a calibration exercise prior to initiate the screening activity. Two investigators will screen all the retrieved citations and abstracts independently to identify eligible articles. Reasons for exclusion will be documented. Full text will be obtained for all eligible titles. Disagreement at any point in the review process will be resolved by discussion. A third party arbitrator will adjudicate disagreements if it is not resolved by discussion. If needed, authors of the studies will be contacted for clarification and additional data. Manual de-duplication will further be performed at this phase as well as the analysis phase. It is implemented by juxtaposing and examining the author names, study or database acronyms, study location and setting, exposure comparisons, sample sizes, and study outcomes of the selected studies. In the case where multiple reports of the same study are identified, they will be coded and linked.

### Data collection process

A pre-tested standardized data extraction form will be used to extract study information. Two reviewers will examine all the eligible articles and extract the data independently. The process will be calibrated between the two investigators. We will attempt to contact the study author(s) twice over 2 weeks by email if additional information is needed to resolve the uncertainties or missing data is important for subsequent analyses. We will impute missing standard deviations or standard errors but not the effect estimates using a previously suggested approach [26].

### Data items

The following information concerning the characteristics and effect estimates of the studies will be collected:

- Publication details (e.g., authors, year of publication, and language);
- Study characteristics (study design, country and setting, sample size, number of sites, follow-up duration, source of funding, method of analysis);
- Characteristics of the study population (e.g., mean age, proportion of male, ethnicity composition, indicators for baseline risk and severity such as proportion of patient with pre-existing heart failure, and TIMI score);
- Details of the exposure under study (e.g., D2B time in term of continuous variable or categorical variables, D2B time categories, and measurement);
- Details of the outcomes of interest (definition, measurement, adjusted and unadjusted effect estimates, confidence interval, and *p* value);
- Variables used for statistical adjustment;
- Variables important to moderator analysis (described later);
- Risk of bias assessment items according to ACROBAT-NRSI.

### Risk of bias in individual studies

All eligible studies will be assessed for risk of bias (RoB) using the Risk Of Bias in Non-randomized Studies - of Interventions (ROBINS-I) tool (previously known as the Cochrane Risk of Bias Assessment Tool: for Non-Randomized Studies of Interventions (ACROBAT-NSRI)) [27]. Two reviewers will perform the assessment independently. Disagreements will be resolved by consensus or by a third reviewer when necessary. The ACROBAT-NSRI: Guidance for using the tool section [27] will be used to establish the framework for RoB assessment specific for this study.

Findings from this assessment will be used in two ways. First, the result will aid in the overall assessment of the quality of the body of evidence. The Grading of Recommendations, Assessment, and Evaluation (GRADE) framework [28] will be used to report the quality of evidence and strength of recommendations. Second, this information will also be used to examine the impact of risk of bias on the findings if deemed feasible. Bias assessment of individual papers will be made available in the final publication, and quality issues of the included studies will be addressed in the discussion section of the study report.

### Data synthesis

We will summarize the study characteristics narratively in the text and/or present the findings in a summary table. We will proceed to synthesize the estimates quantitatively if the following conditions are satisfied:

More than one study are available that pose all the following features:

I. Similar definition and measure for D2B delay (e.g., D2B time as a continuous variable or categorical variable);
II. Negligible difference in the definition and measurement of clinical outcomes (e.g., incidence HF is measured by readmission post-STEMI);
III. Similar study design (e.g., cohort study or case-control study).

Study may be excluded from pooling if the population characteristics and/or the care setting differ significantly from other studies of the same pool. We will report the details and rationale if any individual study is excluded for these reasons.

We will pool studies using random effects meta-analytic models if the above criteria for quantitative synthesis are satisfied. The summary effect measures may include odds ratios, hazard ratios, or relative risk. We will pool and report both the unadjusted and the most adjusted effect sizes for each outcome separately where feasible. Data will be synthesized narratively if quantitative pooling is considered inappropriate. When important heterogeneity precludes pooling, we may still present forest plots without a pooled summary estimate to illustrate the individual study effects.

Statistical heterogeneity will be assessed using Cochrane Q (considered statistically significant at *p* < 0.10) and *I*^2^ statistics. For the interpretation of *I*^2^, we define based on the guidelines given in the Cochrane Handbook for Systematic Reviews of Interventions [29], that 0 to 40 % will be considered as low heterogeneity, 30 to 60 % as moderate heterogeneity, 50 to 90 % will be considered as substantial heterogeneity, and 75 to 100 % as considerable heterogeneity.

### Subgroup analyses and meta-regression

We plan to explore the impact of moderating factors using a combination of subgroup analysis and meta-regression techniques. We will determine the optimal approach for each variable after the exploratory analysis. We will follow previously published guidance [30, 31] for meta-regression if conducted. A random effects meta-regression model will be used. A *p* value <0.10 will signify statistical significance. There should be at least ten studies for a continuous study-level variable if regression analysis is to be considered. For a categorical subgroup variable, each subgroup should have a minimum of four studies. When included studies are mostly small in size, uni-variable meta-regression will be used if the number of studies is insufficient for conducting multivariable analyses. The variables we will be investigating as potential effect modifiers include

I. Data source (data from clinical trials vs. data of clinical registry);
II. Inclusion (vs exclusion) and subgroup analysis of high-risk patients, e.g., patients with cardiogenic shock;
III. Early (symptom onset to hospital arrival of <2 h) vs late presenters (≥2 h);
IV. The completeness of adjustment of confounding factors.

Variables I–II correspond to examining hypothesis 2a (previously stated). We believe that data from clinical trials are different from those from clinical registry as high-risk patients are often excluded in clinical trials. Performing analysis based on different data sources may help us to understand the effect of D2B delay indirectly in patient with varying risk. Variable III corresponds to hypothesis 2c, variable IV to hypothesis 2d. Hypothesis 2b will be examined within the small study effect assessment (described below).

### Sensitivity analysis

We will assess the sensitivity of our estimates by restricting the analyses to studies of low risk of bias and any decisions made regarding data handling.

### Assessment of small study effect

We will evaluate for the presence of small study effect visually using funnel plot if more than ten studies are available. If asymmetry of the funnel plot is suspected, we will attempt to distinguish the possible underlying reasons; when needed, domain expert(s) will be consulted. The decision to test for funnel plot asymmetry will be made after visual inspection. We will follow the recommendations [32] on testing for funnel plot asymmetry for continuous outcomes and dichotomous outcomes. Fixed and random effects estimates of the effect of D2B delay will be compared if between-study heterogeneity is evidenced (*I*^2^ > 0).

### Confidence in cumulative evidence

We will rate the quality of evidence and grade the strength of evidence using the GRADE guideline. The assessment will be performed on each outcome under study separately [28].

### Protocol amendments

This protocol does not update any previously conducted systematic review. Amendments to this protocol will be documented separately with date, description, and rationale and be outlined in the review’s manuscript.

## Discussion

This systematic literature synthesis will address several key questions important to the understanding of the recent negative findings [15, 16] concerning the D2B delay and STEMI outcomes at the population level. Answers to these questions could clarify some controversies discussed above and offer empirical support for or against the hypotheses suggested by earlier analyses. It will also strengthen the validity over some similar efforts previously undertaken.

## Abbreviations

ACROBAT-NSRI, Cochrane Risk of Bias Assessment Tool: for Non-Randomized Studies of Interventions; AMI, acute myocardial infarction; D2B, door-to-balloon; GRADE, Grading of Recommendations, Assessment, and Evaluation; HF, heart failure; PCI, percutaneous coronary intervention; RoB, risk of bias; ROBINS-I, Risk Of Bias in Non-randomized Studies - of Interventions

## Additional files

Additional file 1: Search strategy. (PDF 190 kb) Additional file 2: PRISMA-P checklist. (PDF 224 kb)

## References

[1] White HD, Chew DP (2008). Acute myocardial infarction. Lancet (London, England).

[2] Global Burden of Disease Study C (2015). Global, regional, and national incidence, prevalence, and years lived with disability for 301 acute and chronic diseases and injuries in 188 countries, 1990–2013: a systematic analysis for the global burden of disease study 2013. Lancet (London, England).

[3] O’Gara PT, Kushner FG, Ascheim DD, Casey DE, Chung MK, de Lemos JA, Ettinger SM, Fang JC, Fesmire FM, Franklin BA (2013). ACCF/AHA guideline for the management of ST-elevation myocardial infarction: executive summary: a report of the American College of Cardiology Foundation/American Heart Association Task Force on Practice Guidelines: developed in collaboration with the American College of Emergency Physicians and Society for Cardiovascular Angiography and Interventions. Catheter Cardiovasc Interv.

[4] Wang TY, Peterson ED, Ou FS, Nallamothu BK, Rumsfeld JS, Roe MT (2011). Door-to-balloon times for patients with ST-segment elevation myocardial infarction requiring interhospital transfer for primary percutaneous coronary intervention: a report from the national cardiovascular data registry. Am Heart J.

[5] Braunwald E (1993). The open-artery theory is alive and well—again. N Engl J Med.

[6] Reimer KA, Lowe JE, Rasmussen MM, Jennings RB (1977). The wavefront phenomenon of ischemic cell death.

[7] Kloner RA, Ellis SG, Lange R, Braunwald E (1983). Studies of experimental coronary artery reperfusion. Effects on infarct size, myocardial function, biochemistry, ultrastructure and microvascular damage. Circulation.

[8] The GUSTO Angiographic Investigators (1993). The effects of tissue plasminogen activator, streptokinase, or both on coronary-artery patency, ventricular function, and survival after acute myocardial infarction. N Engl J Med.

[9] Brodie BR, Stone GW, Cox DA, Stuckey TD, Turco M, Tcheng JE, Berger P, Mehran R, McLaughlin M, Costantini C (2006). Impact of treatment delays on outcomes of primary percutaneous coronary intervention for acute myocardial infarction: analysis from the CADILLAC trial. Am Heart J.

[10] McNamara RL, Wang Y, Herrin J, Curtis JP, Bradley EH, Magid DJ, Peterson ED, Blaney M, Frederick PD, Krumholz HM (2006). Effect of door-to-balloon time on mortality in patients with ST-segment elevation myocardial infarction. J Am Coll Cardiol.

[11] Lambert L, Brown K, Segal E, Brophy J, Rodes-Cabau J, Bogaty P. Association Between Timeliness of Reperfusion Therapy and Clinical Outcomes in ST-Elevation Myocardial Infarction. JAMA. 2010;303(21):2148-2155. doi:10.1001/jama.2010.712. 10.1001/jama.2010.712 20516415

[12] Block PC. A campaign to improve the timeliness of primary percutaneous coronary intervention—door-to-balloon: an alliance for quality… Harlan M. Krumholz, MD, SM, FACC; Elizabeth H. Bradley, PhD; Brahmajee K. Nallamothu, MD, FACC; Henry H. Ting, MD, FACC; Wayne B. Batchelor, MD, FACC; Eva Kline-Rogers, RN, MS; Amy F. Stern; Jason Byrd; John E. Brush, Jr., MD, FACC. ACC Cardiosource Review Journal 2008, 17(5):49-53. 10.1016/j.jcin.2007.10.006 19393152

[13] Brennan AL, Andrianopoulos N, Duffy SJ, Reid CM, Clark DJ, Loane P, New G, Black A, Yan BP, Brooks M (2014). Trends in door-to-balloon time and outcomes following primary percutaneous coronary intervention for ST-elevation myocardial infarction: an Australian perspective. Intern Med J.

[14] Krumholz HM, Herrin J, Miller LE, Drye EE, Ling SM, Han LF, Rapp MT, Bradley EH, Nallamothu BK, Nsa W (2011). Improvements in door-to-balloon time in the United States, 2005 to 2010. Circulation.

[15] Flynn A, Moscucci M, Share D, Smith D, LaLonde T, Changezi H, Riba A, Gurm HS (2010). Trends in door-to-balloon time and mortality in patients with ST-elevation myocardial infarction undergoing primary percutaneous coronary intervention. Arch Intern Med.

[16] Menees DS, Peterson ED, Wang Y, Curtis JP, Messenger JC, Rumsfeld JS, Gurm HS (2013). Door-to-balloon time and mortality among patients undergoing primary PCI. N Engl J Med.

[17] Nallamothu BK, Normand SL, Wang Y, Hofer TP, Brush JE, Messenger JC, Bradley EH, Rumsfeld JS, Krumholz HM (2015). Relation between door-to-balloon times and mortality after primary percutaneous coronary intervention over time: a retrospective study. Lancet.

[18] Shiomi H, Nakagawa Y, Morimoto T, Furukawa Y, Nakano A, Shirai S, Taniguchi R, Yamaji K, Nagao K, Suyama T (2012). Association of onset to balloon and door to balloon time with long term clinical outcome in patients with ST elevation acute myocardial infarction having primary percutaneous coronary intervention: observational study. BMJ.

[19] Kong PK, Connolly D, Varma C, Lip G, Millane T, Davis R, Ahmad R (2009). High-risk myocardial infarction patients appear to derive more mortality benefit from short door-to-balloon time than low-risk patients. Int J Clin Pract.

[20] Terkelsen CJ, Sørensen JT, Maeng M, Jensen LO, Tilsted HH, Trautner S, Vach W, Johnsen SP, Thuesen L, Lassen JF (2010). System delay and mortality among patients with STEMI treated with primary percutaneous coronary intervention. JAMA.

[21] Antoniucci D, Valenti R, Migliorini A, Moschi G, Trapani M, Buonamici P, Cerisano G, Bolognese L, Santoro GM (2002). Relation of time to treatment and mortality in patients with acute myocardial infarction undergoing primary coronary angioplasty. Am J Cardiol.

[22] Chen HL, Liu K (2015). Effect of door-to-balloon time on in-hospital mortality in patients with myocardial infarction: a meta-analysis. Int J Cardiol.

[23] Goel K, Pinto DS, Gibson CM (2013). Association of time to reperfusion with left ventricular function and heart failure in patients with acute myocardial infarction treated with primary percutaneous coronary intervention: a systematic review. Am Heart J.

[24] Meier B (2001). The first patient to undergo coronary angioplasty—23-year follow-up. N Engl J Med.

[25] McMurray JJ, Adamopoulos S, Anker SD, Auricchio A, Bohm M, Dickstein K, Falk V, Filippatos G, Fonseca C, Gomez-Sanchez MA (2012). ESC guidelines for the diagnosis and treatment of acute and chronic heart failure 2012: the task force for the diagnosis and treatment of acute and chronic heart failure 2012 of the European Society of Cardiology. Developed in collaboration with the Heart Failure Association (HFA) of the ESC. Eur Heart J.

[26] Wiebe N, Vandermeer B, Platt RW, Klassen TP, Moher D, Barrowman NJ (2006). A systematic review identifies a lack of standardization in methods for handling missing variance data. J Clin Epidemiol.

[27] Sterne JAC HJ, Reeves BC on behalf of the development group for ACROBAT- NRSI: A Cochrane Risk Of Bias Assessment Tool: for Non-Randomized Studies of Interventions (ACROBAT- NRSI). In., Version 1.0.0 edn; 2014.

[28] Guyatt G, Oxman AD, Akl EA, Kunz R, Vist G, Brozek J, Norris S, Falck-Ytter Y, Glasziou P, DeBeer H (2011). GRADE guidelines: 1. Introduction-GRADE evidence profiles and summary of findings tables. J Clin Epidemiol.

[29] Higgins JPT GS (2011). Cochrane Handbook for Systematic Reviews of Interventions Version 5.1.0.

[30] Fu R, Gartlehner G, Grant M, Shamliyan T, Sedrakyan A, Wilt TJ, Griffith L, Oremus M, Raina P, Ismaila A (2011). Conducting quantitative synthesis when comparing medical interventions: AHRQ and the Effective Health Care Program. J Clin Epidemiol.

[31] Dias S, Sutton AJ, Welton NJ, Ades AE (2013). Evidence synthesis for decision making 3: heterogeneity—subgroups, meta-regression, bias, and bias-adjustment. Med Decis Making.

[32] Sterne JA, Sutton AJ, Ioannidis JP, Terrin N, Jones DR, Lau J, Carpenter J, Rucker G, Harbord RM, Schmid CH (2011). Recommendations for examining and interpreting funnel plot asymmetry in meta-analyses of randomised controlled trials. BMJ (Clinical research ed).

