# Supplementary material for: The effect of door-to-balloon delay in primary percutaneous coronary intervention on clinical outcomes of STEMI: a systematic review and meta-analysis protocol
Source: Syst Rev. 2016 Aug 2;5:130. doi: 10.1186/s13643-016-0304-7 (PMC4971724; doi:10.1186/s13643-016-0304-7)
Supplement: Additional file 1: — Search strategy. (PDF 190 kb) [file 13643_2016_304_MOESM1_ESM.pdf]

Search Strategy – PubMed (and Cochrane Library)

Search	Query
**#11**	Search ((((("Time Factors"[Mesh]) OR "Time-to-Treatment"[Mesh])) AND "Myocardial Infarction"[Mesh]) AND ((("Angioplasty, Balloon, Coronary"[Mesh]) OR "Myocardial Revascularization"[Mesh]) OR "Percutaneous Coronary Intervention"[Mesh])) AND "humans"[MeSH Terms] Filters: Publication date from 1977/01/01 to 2015/12/31
**#10**	Search ((((("Time Factors"[Mesh]) OR "Time-to-Treatment"[Mesh])) AND "Myocardial Infarction"[Mesh]) AND ((("Angioplasty, Balloon, Coronary"[Mesh]) OR "Myocardial Revascularization"[Mesh]) OR "Percutaneous Coronary Intervention"[Mesh])) AND "humans"[MeSH Terms]
**#9**	Search "humans"[MeSH Terms]
**#8**	Search (("Angioplasty, Balloon, Coronary"[Mesh]) OR "Myocardial Revascularization"[Mesh]) OR "Percutaneous Coronary Intervention"[Mesh]
**#7**	Search "Percutaneous Coronary Intervention"[Mesh]
**#6**	Search "Myocardial Revascularization"[Mesh]
**#5**	Search "Angioplasty, Balloon, Coronary"[Mesh]
**#4**	Search "Myocardial Infarction"[Mesh]
**#3**	Search ("Time Factors"[Mesh]) OR "Time-to-Treatment"[Mesh]
**#2**	Search "Time Factors"[Mesh]
**#1**	Search "Time-to-Treatment"[Mesh]

Search Strategy – EMBASE via Ovid

#	Search
**1**	heart infarction/
**2**	percutaneous coronary intervention/
**3**	transluminal coronary angioplasty/
**4**	2 or 3
**5**	time/
**6**	time to treatment/
**7**	5 or 6
**8**	1 and 4 and 7
**9**	limit 8 to (human and yr="1977 - 2015")

Search Strategy – CINAHL PLUS

Search ID#	Search Terms
**S17**	S4 AND S8 AND S15 ; Limiters- Publication Year: 1977-2015
**S16**	S4 AND S8 AND S15
**S15**	S9 OR S10 OR S11 OR S12 OR S13 OR S14
**S14**	MH "Revascularization"
**S13**	MH "Myocardial Revascularization"
**S12**	MH "Myocardial Reperfusion"
**S11**	MH "Angioplasty"
**S10**	MH "Angioplasty, Balloon"
**S9**	MH "Angioplasty, Transluminal, Percutaneous Coronary"
**S8**	S5 OR S6 OR S7
**S7**	MH "Coronary Disease"
**S6**	MH "Coronary Thrombosis"
**S5**	MH "Myocardial Infarction"
**S4**	S1 OR S2 OR S3
**S3**	MH "Treatment Delay"
**S2**	MH "Time Factors"
**S1**	MH "Time"
