# Supplementary material for: The effect of door-to-balloon delay in primary percutaneous coronary intervention on clinical outcomes of STEMI: a systematic review and meta-analysis protocol
Source: Syst Rev. 2016 Aug 2;5:130. doi: 10.1186/s13643-016-0304-7 (PMC4971724; doi:10.1186/s13643-016-0304-7)
Supplement: Additional file 2: — PRISMA-P checklist. (PDF 224 kb) [file 13643_2016_304_MOESM2_ESM.pdf]

PRISMA-P Check List

		Checked	Source
	**ADMINISTRATIVE INFORMATION**		
1	Title		
a	Identification	√	Title page
b	Update	NA	
2	Registration	√	Page 2, line 37-38
3	Authors		
a	Contact	√	Title page
b	Contributions	√	page 13, line 278-280
4	Amendments	√	Page 12, line 252-255
5	Support		
a	Sources	√	Page 13, line 272-276
b	Sponsor	NA	
c	Role of sponsor/funder	√	Page 13, line 272-276
	**INTRODUCTION**		
6	Rationale	√	Page 3-4, line 47-77
7	Objectives	√	Page 4-5, line 79-96
	**METHOD**		
8	Eligibility criteria	√	Page 6, line 117-123
9	Information sources	√	Page 5-6, line 107-114
10	Search strategy	√	Page 5, line 100-104
11	Study records		
a	Data management	√	Page 7, line 139-144
b	Selection process	√	Page 7-8, line 147-157
c	Data collection process	√	Page 8, line 160-165
12	Data items	√	Page 8-9, line 167-182
13	Outcomes and prioritization	√	Page 6, line 126-136
14	Risk of bias in individual studies	√	Page 9, line 185-196
15	Data		
a	Criteria for proceeding with quantitative synthesis	√	Page 10, line 200-208
b	Planned summary measures, methods of handling data, methods of combining data from studies	√	Page 10-11, line 209-219
c	Proposed additional analysis e.g. sensitivity or subgroup analyses, meta-regression	√	Page 11, line 221-237
d	Type of summary planned if quantitative synthesis not appropriate	√	Page 10, line 212-214
16	Meta-bias(es)	√	Page 12, line 239-246
17	Confidence in cumulative evidence	√	Page 12, line 249-250

NA - Not Applicable
